# Culturally responsive approaches to brain health and dementia education for American Indian, Alaska Native, and Native Hawaiian communities

**DOI:** 10.1093/geront/gnaf233

**Published:** 2025-10-09

**Authors:** Breana Dorame, Kelsey Donnellan, Breannon Babbel, Ron Eppes, Bill Benson, Kendra Kuehn, Megan Dicken, Courtney Hoskins

**Affiliations:** International Association for Indigenous Aging, Silver Spring, Maryland, United States; International Association for Indigenous Aging, Silver Spring, Maryland, United States; International Association for Indigenous Aging, Silver Spring, Maryland, United States; International Association for Indigenous Aging, Silver Spring, Maryland, United States; International Association for Indigenous Aging, Silver Spring, Maryland, United States; International Association for Indigenous Aging, Silver Spring, Maryland, United States; International Association for Indigenous Aging, Silver Spring, Maryland, United States; International Association for Indigenous Aging, Silver Spring, Maryland, United States

**Keywords:** Native elders, Indigenous, Community engagement, Cultural sensitivity

## Abstract

While American Indian, Alaska Native, and Native Hawaiian (AI/AN/NH) populations have disproportionately been affected by dementia, these communities are resilient and offer critical insight about dementia and brain health through the aging process. Existing dementia education often neglects cultural nuances and realities that influence health beliefs and practices in these communities, focusing on disparities or erasing Native experiences entirely. This article highlights the importance and impact of culturally tailoring dementia education, serving as a call to action for providers, policymakers, and partners to consider the needs of AI/AN/NH communities when addressing dementia. As the only Centers for Disease Control and Prevention-funded AI/AN Resource Center for Brain Health, the International Association for Indigenous Aging (IA^2^) has employed a multifaceted, strength-based approach for effectively engaging with and supporting Native populations in addressing disparities in dementia and brain health. This approach enables the co-creation of inclusive, impactful dementia education resources that resonate with AI/AN/NH populations. IA^2^’s work underscores the importance of integrating community engagement, Indigenous knowledge, and traditional practice into public health frameworks to address the complex challenges dementia poses in Native communities.

## Dementia in American Indian, Alaska Native, and Native Hawaiian populations

Dementia and brain health are pressing public health concerns. Rising rates of cognitive decline present significant challenges for healthcare systems and communities. However, the impact of dementia is not experienced equally across all populations. For American Indian, Alaska Native, and Native Hawaiian (AI/AN/NH) communities, the intersection of cultural, historical, and socioeconomic factors shapes how dementia is perceived, understood, and managed. Native people hold deep cultural beliefs about aging, memory, and care for Elders, often rooted in traditions that emphasize the communal role of Elders as knowledge keepers and cultural curators (Crouch et al., 2023). This makes cognitive decline not only a medical issue in Native communities but also a cultural one.

AI/AN/NH populations experience disproportionately high rates of risk factors for dementia, including diabetes and cardiovascular disease, which often emerge earlier in life (Alzheimer’s Association, 2021; Blair et al., 2021; Harris & Jones-Smith, 2020; Sehar et al., 2023). The incidence, prevalence, and severity of these risk factors are further exacerbated by systemic barriers, such as the chronic underfunding of tribal health services, geographic isolation, and limited availability of culturally appropriate care (Jaramillo et al., 2022; Kaiser Family Foundation, 2021; National Indian Health Board, n.d.; U.S. Commission on Civil Rights, 2018). Centuries of colonization, forced assimilation, and systemic oppression, in particular, have disrupted traditional health practices, leading to mistrust in healthcare systems and gaps in services (Brown & Wilson, 2022; Solomon et al., 2022; Warne & Frizzell, 2014). Socioeconomic challenges, such as poverty and limited educational opportunities, further hinder access to preventive care among these populations (Harris & Jones-Smith, 2020; Jernigan et al., 2020). Collectively, these factors contribute to a higher prevalence of dementia and delayed diagnosis after symptom onset among AI/AN/NH populations, in addition to an elevated risk of earlier onset among NH (Alzheimer’s Association, 2021; Blair et al., 2021; Harris & Jones-Smith, 2020; Sehar et al., 2023).

AI/AN/NH peoples’ health literacy and awareness around brain health are often limited due to the historical erasure of Native voices in public health initiatives (Jernigan & Maudrie, 2025). Mainstream dementia-related resources tend to focus on the clinical aspects of care, overlooking the cultural values and community-based practices that are central to Native experiences with aging. Such cultural erasure reduces the relevance of resources for AI/AN/NH people, as they often focus on deficits rather than resilience (Henderson & Henderson, 2002; Lewis, 2019).

Addressing dementia in Native communities requires a culturally sensitive approach that acknowledges these histories and realities (Crouch et al., 2023). The International Association for Indigenous Aging (IA^2^) has responded to this need by creating the only CDC-funded AI/AN Resource Center for Brain Health, focusing on the co-creation of educational resources with Native communities that respect and reflect Indigenous ways of knowing and healing. By tailoring dementia-related information and interventions to the specific cultural contexts of these communities, IA^2^’s efforts serve as a model for how public health initiatives can be made more inclusive and effective.

### The foundation of sovereignty

Sovereignty is a foundational force shaping public health outcomes in AI/AN/NH populations. Addressing dementia, including prevention, early detection, and care, requires incorporating sovereignty into all aspects of public health research, policy, and resource development (Cohen, 2012; National Congress of American Indians, n.d.).

For aging AI/AN/NH populations, sovereignty is not only political; it is also deeply connected to cultural preservation and the well-being of Native Elders. Native Elders play a crucial role in maintaining traditional knowledge, language, and cultural practices, all of which are foundational to Native identity. Sovereignty allows these communities to control their healthcare systems and social services, ensuring that Elder care is culturally appropriate and community-driven (IA^2^, 2023; Jernigan et al., 2020).

Although sovereignty is a shared principle, its expression varies significantly across AI/AN/NH groups due to distinct historical, legal, and political contexts. Acknowledging these unique expressions of sovereignty is vital for developing public health strategies. For federally recognized American Indian tribes in the lower 48, sovereignty is rooted in their status as pre-existing political entities, affirmed through treaties and federal Indian law (Worcester v. Georgia, 1832). Alaska Native governance stems from the Alaska Native Claims Settlement Act of 1971 and is exercised largely through regional and village-based corporations rather than land-based tribal governments (Cohen, 2012; Lewis, 2019). In contrast, Native Hawaiian sovereignty originated as a result of the overthrow and annexation of the Hawaiian Kingdom, with ongoing movements for self-determination and legislative efforts seeking to restore some level of governance (Apology Resolution, 1993; Liliʻuokalani, 1898).

Sovereignty is a living, evolving principle central to ongoing efforts to safeguard AI/AN/NH communities and Elders. Honoring sovereignty is fundamental for creating culturally grounded, community-led, and sustainable dementia care and education. Interventions that fail to recognize this foundation risk imposing external frameworks that may disregard Indigenous knowledge systems, leading to ineffective or harmful outcomes (IA^2^, 2023; Jernigan et al., 2020). Thus, centering sovereignty in dementia care and education allows for community-driven solutions that honor tradition, build trust, and foster healthier futures for AI/AN/NH populations (IA^2^, 2023; CDC, 2020).

## Tailored messaging in dementia education

Tailoring dementia-related information for AI/AN/NH populations is critical for promoting effective care and intervention strategies that resonate within these communities. This requires integrating historical, political, and cultural contexts into the development of resources, something that conventional public health strategies often fail to do.

Mainstream approaches frequently overlook the disproportionately high rates of chronic disease in these populations, as well as the enduring impacts of colonization, systemic underfunding, and deep-seated mistrust in healthcare systems (John-Henderson et al., 2023; Warne et al., 2025). Moreover, conventional strategies often rely on deficit-based messaging without acknowledging Indigenous knowledge systems, caregiving traditions, and the cultural significance of aging, further ­widening gaps in health literacy, engagement, and access (Dickerson et al., 2020; Kaholokula et al., 2025; Warne et al., 2025).

In contrast, culturally tailored resources can produce more effective health interventions, ensuring that health information aligns with traditional beliefs, values, and community practices (Goins et al., 2011; Martindale-Adams et al., 2017; Pedersen et al., 2022). This fosters greater acceptance, trust, and engagement with public health messaging. In many Native communities, memory loss and aging are viewed through a lens of spiritual and communal significance rather than solely as a clinical condition (Pace & Grenier, 2017). Failure to acknowledge these perspectives in public health messaging can alienate individuals from seeking care or support.

Incorporating Native ways of knowing—such as storytelling, traditional caregiving practices, and community-driven solutions—not only enhances the relevance of dementia-related interventions but also addresses gaps in health literacy. Culturally tailored resources promote a strength-based approach, highlighting the resilience and adaptive strategies within Native communities, rather than focusing solely on deficits or disparities (Blair et al., 2021; Jernigan et al., 2020; Kaholokula et al., 2025; Masaki et al., 2017; Mokuau & Braun, 2007).

Upholding sovereignty and centering culture are foundational in reversing ineffective public health strategies. Empowering Native communities to lead and design brain health and dementia care efforts ensures that strategies are aligned with their values, languages, and ways of life (IA^2^, 2023; National Congress of American Indians, n.d.). Key strategies to effectively tailor materials include the use of culturally relevant imagery and plain language.

### Culturally relevant imagery

Imagery in health information plays a crucial role in connecting with Native communities, particularly when addressing dementia and brain health among AI/AN/NH populations. Images can resonate with cultural significance, helping to communicate complex or sensitive health topics in approachable and relatable ways (Public Health Institute, 2021; Stephens et al., 2020). For instance, using imagery that reflects the landscapes, symbols, and people familiar with these communities fosters a sense of inclusion, reinforcing that these resources are made specifically for their communities, which can increase engagement and trust in the material.

For dementia and brain health resources, culturally relevant imagery is especially valuable because these issues can carry unique meanings within Native contexts. Visuals that showcase multigenerational family connections or reflect traditional knowledge and practices are powerful for educating about brain health within a framework of cultural continuity. When Native people see their values and lifestyles represented in health resources, it enhances understanding and reduces stigma, helping to bridge potential gaps in health literacy and making it easier to discuss sensitive topics like cognitive decline. Culturally aligned imagery supports respectful communication and affirms the shared experiences of Indigenous individuals and communities, ultimately promoting effective public health outreach.

### Plain language

Similarly, using plain language in the development of Native-tailored brain health resources is critical in improving the accessibility and cultural appropriateness for AI/AN/NH populations. In addition to reducing stigma, using plain language also promotes health literacy, community empowerment, communication, and engagement—all of which can lead to improved health outcomes overall.

For diverse AI/AN/NH communities, plain language is essential for making complex health information accessible to individuals with varying literacy, education levels, and linguistic backgrounds. Since community members may be unfamiliar with medical terminology, using clear, straightforward language helps ensure everyone can comprehend essential health messages (CDC, 2024; Jernigan et al., 2020). Developing plain language materials in consultation with Native communities also incorporates cultural nuances and reflects traditional values, enhancing relatability, trust, and a sense of ownership over the information provided (Lewis, 2019; Mokuau & Braun, 2007). Improving accessibility and cultural appropriateness, in turn, improves health literacy by helping communities understand risks, prevention measures, and available resources. Supporting informed decision-making about brain health and dementia care is an important factor in better health outcomes (Blair et al., 2021; Henderson & Henderson, 2002).

Plain language also plays a critical role in reducing stigma around conditions like dementia, which can be difficult to discuss in some communities. Clear, compassionate messaging can promote understanding and encourage open discussions about brain health, strengthening support networks for affected individuals and families (Harris & Jones-Smith, 2020). Additionally, when health information is easy to understand, individuals are empowered to take action such as seeking preventive care, participating in programs, or sharing knowledge with others (CDC, 2024; Geana et al., 2012; Jernigan et al., 2020). This improves communication between providers and community members and enhances engagement in screenings, workshops, and services.

Ultimately, culturally tailoring brain health resources through the use of imagery and plain language improves understanding, promotes empowerment, and supports community-driven solutions that build trust and strengthen relationships, all of which lead to improved public health outcomes for AI/AN/NH populations (IA^2^, 2023; Masaki et al., 2017).

## IA^2^’s role: the CDC-funded AI/an resource center for brain health

The International Association for Indigenous Aging serves as a national hub for dementia and brain health information for AI/AN populations. As a funded grant recipient under the National Healthy Brain Initiative cooperative agreement, IA^2^ has developed a multifaceted approach to its brain health work that exemplifies how the core principles of sovereignty and tailored messaging can be meaningfully and practically implemented across dementia-related outreach, education, and resource development. This approach includes community engagement and capacity building, focusing on culturally tailored resources, technical assistance, and training for tribal leadership, public health staff, and Native Elder services advocates. IA^2^’s work highlights the importance of culturally relevant public health strategies in addressing dementia disparities within Native populations (IA^2^, 2023).

Partnership and community engagement have been especially instrumental in raising awareness about dementia risk reduction, early detection, and the importance of caregiving support in Native populations. By partnering directly with Tribal Nations and Native-serving organizations—including Title VI Directors, Tribal Aging Services, Indian Health Organizations and Health Boards—IA^2^ continues to ensure that dementia education upholds sovereignty and is culturally appropriate, addressing the specific needs of Native Elders and their families.

One of IA^2^’s most significant contributions to Indigenous brain health education is its leadership in the development of the *Healthy Brain Initiative Road Map for American Indian and Alaska Native Peoples*. This national-level framework emerged from an inclusive, Indigenous-led process of webinars, listening sessions, and focus groups with Native health and aging service leaders. The Roadmap outlines actionable, culturally informed strategies for promoting brain health and dementia care, with a focus on community empowerment and health equity. The initiative exemplifies both sovereignty in action—by centering Native leadership in strategic planning—and tailored messaging, by ensuring that the recommended actions are grounded in Indigenous cultural frameworks. To expand the cultural relevancy of its existing resources, IA^2^ is currently developing the Native Hawaiian Road Map to address brain health, further reflecting IA^2^’s commitment to acknowledging the diversity of Indigenous experiences and honoring the sovereignty of each group.

Beyond the Roadmap, IA^2^ maintains one of the most robust online resource libraries of culturally tailored, Indigenous-focused brain health and dementia resources, available both online and in print at no cost to Native communities (see online Supplementary material). To maintain the currentness of its catalogue, IA^2^ conducts community and partnership engagement through an Executive Steering Committee, a National Advisory Group, Title VI, and community members through a variety of avenues such as listening sessions and talking circles, periodic CDC review, and plain language assessments. This ensures that the materials remain responsive to evolving community needs, reflecting IA^2^’s commitment to improving dementia care through culturally sensitive and community-driven approaches (IA^2^, 2023).

## Recommendations for culturally responsive dementia education

The principles of sovereignty and tailored messaging form the foundation of effective public health engagement for promoting dementia education within AI/AN/NH communities. This requires an approach grounded in cultural sensitivity, community engagement, and respect for sovereignty. The following recommendations offer a starting point for developing a culturally responsive approach to brain health and dementia education.

### Native resiliency

AI/AN/NH communities have long embraced cultural practices that promote mental and emotional well-being as they age. Traditional knowledge—such as storytelling, communal activities, and spiritual practices—plays a critical role in fostering cognitive engagement and social connectedness among Elders. As cultural stewards, Elders’ active involvement in community life protects against isolation and cognitive decline. Despite continued systemic challenges, these communities continue to adapt their traditional practices to modern health concerns, demonstrating strength and resiliency in managing brain health across generations. Shifting from a deficit-based approach to a strengths-based one promotes optimal health and well-being within Native communities by centering resilience, cultural knowledge, and community assets (John-Henderson et al., 2023).

### Community engagement

Community engagement is vital to the success of culturally responsive dementia education. IA^2^’s work demonstrates how partnerships built on respect and cultural knowledge foster trust and program effectiveness. Engagement strategies, including talking circles and listening sessions, facilitate direct feedback with Elders, caregivers, and local health workers to promote better health outcomes by enhancing health program uptake and sustainability.

### Cultural sensitivity

Understanding and respecting the cultural values, beliefs, and practices of Native people helps bridge the gap between Western healthcare approaches and Native perspectives on aging. When health services reflect cultural traditions and address the lived experiences of Native Elders, communities are more likely to engage with care and adopt preventative practices. This sensitivity strengthens the relationship between healthcare providers and Native communities, fostering long-term partnerships and trust.

**Figure 1.**
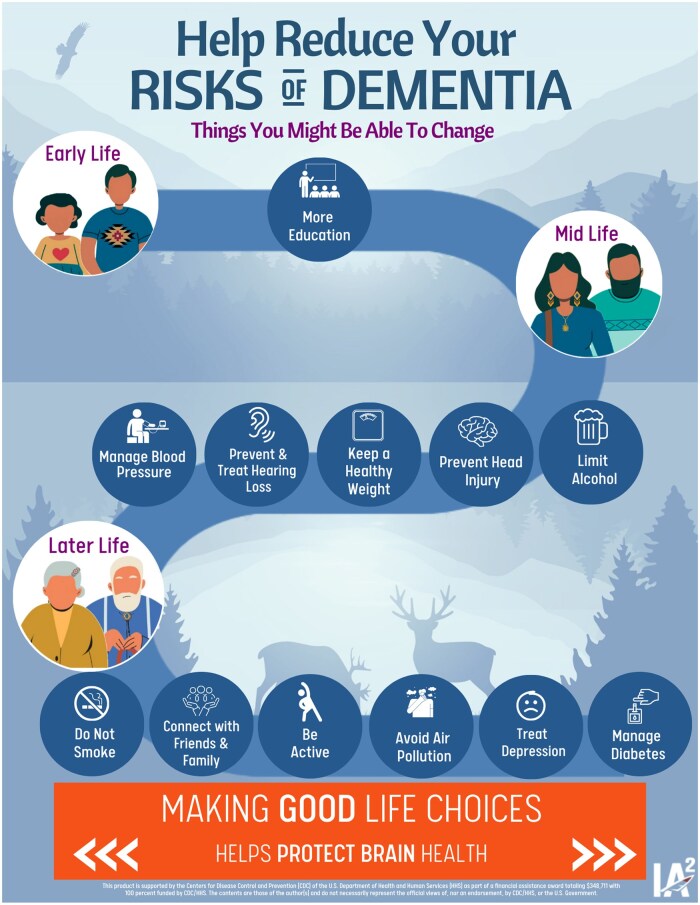
AI/AN (American Indian, Alaska Native)-tailored Lancet risk factors for dementia, adapted by IA^2^.

The International Association for Indigenous Aging’s dementia education efforts include a strong commitment to Native resiliency, community engagement, and cultural sensitivity. For example, the development of the *Dementia Rick Reduction Graphic* (Figure 1), reflected a highly collaborative process, incorporating talking circles and input from tribal members, dementia advocates, and university partners. Similarly, the *Dementia Friends for AI/AN Communities* initiative adopts a train-the-trainer model, empowering local “Champions” to raise dementia awareness and share culturally relevant information within their own communities. IA^2^’s *Healthy Brain Rack Card* further reflects cultural sensitivity by addressing the long-term impact of historical trauma—such as forced relocation and disrupted food systems—while emphasizing the strengths and resilience inherent in Native communities. Together, these efforts demonstrate a model of dementia education driven by communities, informed by culture, and supportive of sovereignty.

These strategies underscore the importance of resilience, community engagement, and cultural sensitivity in addressing brain health within Native populations. IA^2^ prioritizes initiatives that elevate the voices of Native Elders, caregivers, and tribal leaders throughout every stage of program design and implementation. By weaving cultural relevance into every aspect of its work—from visual design and tailored messaging to Indigenous governance and delivery—IA^2^’s work upholds sovereignty and reflects the diverse needs AI/AN/NH communities’ efforts. This approach, epitomized in the CDC Healthy Brain Initiative Component B Grant, not only strengthens dementia care but also fosters a sense of empowerment and self-determination that is essential for long-term health outcomes.

## Call to action

Native-serving organizations, providers, policymakers, and stakeholders play a vital role in shaping the future of brain health and dementia care for AI/AN/NH communities. As the number of Native Elders and their families affected by dementia continues to grow, so does the need for culturally grounded approaches that reflect Indigenous worldviews, honor traditional knowledge, and support community-driven solutions. This call to action highlights how organizations, communities, and tribal partners can create, promote, and sustain culturally responsive dementia and brain health care for AI/AN/NH communities.

### Healthcare providers

Healthcare providers serving AI/AN/NH populations must prioritize culturally responsive care and education that integrates traditional knowledge and practices. Culturally sensitive dementia care and brain health services should reflect Native perspectives on aging, health, and wellness, incorporating practices like storytelling, community involvement, and spiritual healing. Healthcare providers can also adapt culturally sensitive dementia care and brain health services for Native people living in urban settings, taking into account their unique experiences.

Many urban Native individuals may feel disconnected from traditional lands and communities, and healthcare providers should incorporate cultural knowledge, such as storytelling, family involvement, and community gatherings, into dementia care practices to bridge this gap. Providers should actively seek to understand the cultural values of the populations they serve and work alongside traditional healers and community members to build trust and deliver care that resonates with these communities. This approach not only improves health outcomes but also respects the cultural integrity of Native populations.

### Policymakers

Policymakers must advocate for policies that prioritize the needs of AI/AN/NH communities in dementia care and brain health services. Legislation should address the unique challenges faced by these populations, including geographic isolation, limited access to healthcare, and the cultural dissonance inherent in mainstream medical approaches. Policies should support the development and funding of culturally tailored resources and encourage the inclusion of Native voices in public health planning. By integrating Native concerns and perspectives into broader dementia care initiatives, policymakers can promote equity and improve the health of Native Elders.

### Partners and stakeholders

Partners and stakeholders, including public health organizations, researchers, and nonprofit agencies, are encouraged to collaborate directly with tribal communities to co-create meaningful, culturally relevant dementia-related resources. Engaging Native leaders, caregivers, urban Native people, and community members in the development process ensures that materials reflect lived experiences and community priorities. Stakeholders should build partnerships based on mutual respect and a shared commitment to improving the quality of care for Native Elders. Collaborative efforts can result in more effective interventions, greater trust, and lasting improvements in dementia care within Native communities.

This call to action highlights the crucial role of healthcare providers, policymakers, and stakeholders in addressing the increasing needs of Native Elders in dementia care, underscoring the importance of culturally responsive and community-driven solutions. Leveraging community strengths, engaging Native voices, and upholding sovereignty can drive lasting change that supports the well-being and dignity of current and future generations.

## Conclusion

This article highlights how IA^2^’s work represents a critical advancement in addressing the unique challenges AI/AN/NH populations face in managing dementia and promoting brain health. By integrating Indigenous knowledge systems, community engagement, and traditional practices into modern public health frameworks, IA^2^ has effectively created resources that resonate with Native communities through a strength-based approach, fostering greater acceptance and proactive management of brain health.

As dementia rates continue to rise in AI/AN/NH populations, public health strategies must honor sovereignty by providing autonomy and support to develop solutions that align with their values and traditions. To achieve this, healthcare providers, policymakers, and public health stakeholders must recognize the central role that culture and community play in the aging process for Native communities. IA^2^’s contributions offer a model for how public health can effectively engage Native populations, ensuring that future initiatives are inclusive and impactful. IA^2^’s success underscores the potential of collaborative, culturally responsive interventions to address health disparities and build resilience to support healthy aging among AI/AN/NH populations. Continued partnerships between public health organizations, tribal leaders, and community members will be vital in fostering dementia education that is inclusive and impactful.

## Supplementary Material

gnaf233_Supplementary_Data

## Data Availability

This article does not report data and therefore the preregistration and data availability requirements are not applicable.
