# Supplementary material for: Culturally responsive approaches to brain health and dementia education for American Indian, Alaska Native, and Native Hawaiian communities
Source: Gerontologist. 2025 Oct 9;65(Suppl 1):S53–9. doi: 10.1093/geront/gnaf233 (PMC12736973; doi:10.1093/geront/gnaf233)
Supplement: gnaf233_Supplementary_Data [file gnaf233_supplementary_data.docx]

IA2 Online Supplemental Materials

IA^2^ provides a wide range of culturally tailored resources to support brain health and dementia awareness within AI/AN/NH communities. IA^2^ continues to update the resources through feedback from the Executive Steering Committee, the National Advisory Group, Title VI, and Community Members through listening sessions and talking circles, CDC review, plain language assessment, and more. Some of the key resources include:

[**Healthy Heart Healthy Brain**](https://iasquared.org/resources/dementia-risk-reduction-flyers-healthy-heart-healthy-brain/) **-** These materials focus on reducing dementia risks through lifestyle changes that IA^2^ specifically adapted for Native communities. Topics include managing blood pressure, staying socially active, and limiting alcohol​. The Healthy Heart Healthy Brain flyers and posters are a quick reference for community members to learn about the connection between heart and brain health (IA², 2023).

[**10 Signs of Thinking or Memory Changes**](https://iasquared.org/resources/10-signs-of-thinking-or-memory-changes-that-might-be-dementia/) **–** A flyer highlighting early dementia signs, available in various formats with customization options for tribal communities. This flyer reiterates typical signs of aging versus changes in memory or thinking that are not typical signs of aging or could be early signs of dementia. There are two design options, one with pictures and one with no pictures. There are currently 5 design options with different images at the top for the design with pictures (IA², 2023).

[**Healthy Food, Healthy Brain Rack Card Series**](https://iasquared.org/resources/healthy-food-healthy-brain-rack-card-series/) – This series includes culturally relevant dietary advice to promote brain health and is designed for distribution through various community channels like senior centers and tribal media​. This healthy eating-focused message series contains practical advice and culturally relevant recipes. Tribal meal delivery programs can incorporate weekly distribution for in-person and home-delivered meals (IA², 2023).

[**Healthy Brain Rack Card**](https://iasquared.org/resources/healthy-brain-rack-card/) **–** The Healthy Brain Rack Card provides essential information to help AI/AN communities reduce their risk of dementia. Highlighting how historical traumas like the loss of land, forced relocation, and food transitions increase the risks of dementia in these populations, the card also offers strategies for promoting brain health. The rack card emphasizes that AI/AN communities have many strengths to draw upon for brain health and provides links to resources from IA², the Alzheimer’s Association, and Indian Health Services (IA², 2023).

[**Dementia 101**](https://youtu.be/j2dfXUktXcM) **–** IA^2^ created and released a six-minute video on Dementia 101 for AI/AN communities. This video provides information about dementia and how it pertains to AI/AN communities. The video can act as a stand-alone educational piece or as a partner piece with Dementia Bingo. The video is intended for the public to use (IA², 2023).

[**Dementia Bingo**](https://iasquared.org/resources/dementia-bingo/) – A cognitive activity designed to support individuals with dementia, offering both entertainment and cognitive stimulation. It serves as an intervention tool for caregivers and people living with dementia, offering emotional respite and cognitive stimulation.  Individual game sessions are organized around dementia-relevant topics to enhance meaningful engagement (IA², 2023).

[**Tribal Resolution Toolkit**](https://iasquared.org/resources/tribal-resolution-toolkit/#1674247993922-847cdc97-324da606-274d) **–**The sample resolution is a mechanism for a tribal governing body to address program and/or policy matters that affect the welfare of Native governments and communities, specifically those affected by Alzheimer’s disease and related dementias (ADRD). The language of the resolution should reflect tribal governments’ preferred words and identification of their people. We encourage the tribal governing body to modify the sample resolution to tailor it for their community (IA², 2023).

[**ADRD State Plans**](https://iasquared.org/resources/adrd-state-plans/) **–** This guide is a resource to aid in ADRD policy and planning for AI/AN tribal communities, their leaders, and state and local public health entities. IA^2^ identified language about ADRD and engagement practices referencing AI/AN populations and communities in 18 existing states’ Alzheimer’s plans. This language forms the context for and the structure of this guide. This document contains word-for-word excerpts (only edited for format) from current state Alzheimer’s and dementia plans that are inclusive of “American Indian” and/or “Alaska Native” and other culturally relevant terms (see methods section). While these 18 plans include some type of culturally inclusive language or reference, only 8 of the 18 plans include population-specific language. Specific dementia topics organize content, and an index of high-frequency keywords and phrases is included.​

This document does not provide direct guidance or compile best practices. Instead, it indexes current language in existing state plans that may be useful in considering future modifications to such state Alzheimer’s and dementia plans, the development of new state ADRD plans, or the creation of tribal-specific plans. The current language can also be helpful for other efforts to include Native peoples in Alzheimer’s and dementia-related strategic products (IA², 2023).

[**Dementia Risk Reduction Graphic**](https://iasquared.org/resources/help-reduce-your-risks-of-dementia/) **-** IA² has also adapted [**The Lancet Risk Factors for**](https://www.thelancet.com/infographics-do/dementia-risk) **Dementia** (see Figure 1) infographic and created a version culturally tailored towards American Indian and Alaska Native communities. The process of creating this infographic included talking circles with tribal community members, [**Dementia Friends & Champions**](https://iasquared.org/dementia-friends/), [**the University of Nevada**](https://www.unr.edu/public-health/centers/dementia-engagement-education-and-research-program) [**(DEER) Program**](https://www.unr.edu/public-health/centers/dementia-engagement-education-and-research-program), [**IA²’s Brain Health Leadership team**](https://iasquared.org/brain-health/brain-health-leadership/), and internal IA² staff members. The infographic is also in the Dementia Friends workbook for AI and AN Communities. Users can download the infographic as a standalone document on the IA² website or use the infographic as part of the Dementia Friends for AI and AN Communities workbook (IA², 2023).

[**Dementia Friends American Indian and Alaska Native Communities**](https://iasquared.org/dementia-friends/) **-** Dementia Friends is a global movement focused on transforming perceptions of dementia by educating communities on what dementia is and how it affects individuals. IA², in collaboration with Dementia Friendly America Nevada and key community members, has adapted Dementia Friends materials specifically for AI/AN individuals and communities. Recognizing the distinct needs and cultural contexts of Native communities, IA² has worked to ensure these resources are relevant, respectful, and supportive.

The ultimate goal of Dementia Friends is to create dementia-friendly communities where those living with dementia or memory loss, along with their families and community members, can thrive. For IA², the approach to achieving this goal is through a “ripple effect” that begins with their specialized Dementia Friends for American Indian and Alaska Native Communities Information Session and Champion Training. By training community members to become “Champions,” IA² empowers them to share knowledge, resources, and support within their communities. Application of Dementia Friends training creates a network of dementia-friendly advocates who understand Indigenous communities' cultural values and caregiving traditions, fostering a supportive environment for individuals with dementia and promoting greater awareness and understanding throughout the community (IA², 2023).
